# Rituximab in steroid-sensitive nephrotic syndrome: lessons from clinical trials

**DOI:** 10.1007/s00467-017-3746-9

**Published:** 2017-07-17

**Authors:** Kazumoto Iijima, Mayumi Sako, Koichi Kamei, Kandai Nozu

**Affiliations:** 1 Department of Pediatrics, Kobe University Graduate School of Medicine, 7-5-2 Kusunoki-cho, Chuo-ku, Kobe, 650-0017 Japan; 2 Division for Clinical Trials, Department of Clinical Research, Center for Clinical Research and Development, National Center for Child Health and Development, Tokyo, Japan; 3 Division of Nephrology and Rheumatology, National Center for Child Health and Development, Tokyo, Japan

**Keywords:** Frequently relapsing nephrotic syndrome, Steroid-dependent nephrotic syndrome, Rituximab, Randomized, Clinical trial, Clinical management

## Abstract

Idiopathic nephrotic syndrome is the most common chronic glomerular disease in children. A total of 80–90% of patients with childhood idiopathic nephrotic syndrome achieve remission with steroid therapy [steroid-sensitive nephrotic syndrome (SSNS)]. However, approximately 50% of children with SSNS develop frequently relapsing nephrotic syndrome (FRNS) or steroid-dependent nephrotic syndrome (SDNS). Children with FRNS or SDNS are usually treated with immunosuppressive agents, but 10–20% of children receiving immunosuppressive agents still show frequent relapses or steroid dependence during or after treatment, defined as complicated FRNS or SDNS. Rituximab, a chimeric anti-CD20 monoclonal antibody that was originally developed to treat patients with B-cell non-Hodgkin’s lymphoma, is currently used for treating SSNS. In this review we highlight recent studies, mainly randomized controlled trials of rituximab for SSNS, including complicated and uncomplicated forms of FRNS or SDNS in children. We also discuss the effects of these studies on the management of patients suffering from these conditions.

## Introduction

Idiopathic nephrotic syndrome is the most common chronic glomerular disease in children, occurring in two of 100,000 children per year in Caucasians [1]. Japanese children develop this disease at a three- to four-fold higher frequency than Caucasians [2]. A total of 80–90% of patients with childhood idiopathic nephrotic syndrome achieve remission with steroid therapy [steroid-sensitive nephrotic syndrome (SSNS)] [3]. Approximately 50% of children with SSNS develop frequently relapsing nephrotic syndrome (FRNS), which is defined as at least four relapses per year or at least two relapses within 6 months of the initial presentation. A total of 50–60% of children with FRNS develop two consecutive relapses during tapering or within 14 days of stopping steroid therapy, which is referred to as steroid-dependent nephrotic syndrome (SDNS) [4]. Standard treatments for FRNS or SDNS in children are immunosuppressive agents, including cyclophosphamide, chlorambucil, cyclosporine (CyA), tacrolimus and levamisole [5]. The Kidney Disease: Improving Global Outcomes (KDIGO) Clinical Practice Guideline for Glomerulonephritis recommends treatment with alkylating agents (such as cyclophosphamide or chlorambucil), levamisole, calcineurin inhibitors (including CyA or tacrolimus) and mycophenolate mofetil (MMF) as corticosteroid-sparing agents for children with FRNS or SDNS [6]. The 2013 Clinical Practice Guidelines for Pediatric Nephrotic Syndrome of the Japanese Society for Pediatric Nephrology recommend CyA, cyclophosphamide or mizoribine as drug therapy for children with FRNS or SDNS [7]. Although these treatments are generally successful in most patients, at least 10–20% of children receiving immunosuppressive agents still show frequent relapses or steroid dependence during or after treatment. Additionally, some patients with steroid-resistant nephrotic syndrome (SRNS) develop steroid-sensitive frequent relapses or steroid dependence after achieving complete remission by immunosuppressive therapies, including calcineurin inhibitors. A 5-year follow-up study of CyA treatment in children with SRNS showed that seven of 31 (23%) patients developed frequent relapses under immunosuppressive therapy after achieving complete remission [8]. Therefore, these children require long-term steroid treatment, even though they experience serious side effects from the drug. We have defined these conditions as complicated FRNS or SDNS [9–11]. In this review we also define FRNS or SDNS that has never been treated with immunosuppressive agents as uncomplicated FRNS or SDNS.

Rituximab is a chimeric anti-CD20 monoclonal antibody that inhibits CD20-mediated B-cell proliferation and differentiation. It was originally developed to treat patients with B-cell non-Hodgkin’s lymphoma. This monoclonal antibody is currently used for treating various autoimmune diseases, such as rheumatoid arthritis, Wegener’s granulomatosis and microscopic polyangiitis. In this review we highlight recent studies, mainly randomized, controlled trials (RCTs) of rituximab for SSNS, including complicated and uncomplicated forms of FRNS or SDNS, in children. We also discuss the effects of these studies on the management of patients suffering from these conditions.

## Case reports, case series and survey studies of rituximab for complicated FRNS/SDNS

Benz et al. reported that four doses of rituximab treatment induced long-term remission of complicated SDNS and idiopathic thrombocytopenic purpura in a 16-year-old boy [12]. This is the first report to suggest the efficacy of rituximab for treating complicated FRNS/SDNS. Gilbert et al. and Francois et al. reported that four doses of rituximab were effective for maintaining remission in patients with childhood-onset complicated FRNS/SDNS [13, 14]. Smith reported that a boy suffering from complicated FRNS/SDNS for longer than 11 years achieved 10 months of remission after a single rituximab infusion [15].

Following those case reports, Guigonis et al. reported a multicenter case series (*n* = 22) including patients who received two to four doses of rituximab as treatment of severe steroid- or cyclosporine-dependent nephrotic syndrome (complicated SDNS) [16]. One or more immunosuppressive treatments could be withdrawn in 19 (85%) patients, with no relapse of proteinuria and without an increase in other immunosuppressive drugs. Rituximab was effective in all of the patients when administered during a proteinuria-free period in association with other immunosuppressive drugs. Adverse effects were observed in 45% of cases, but most of these were mild and transient. Gulati et al. showed that two doses of rituximab treatment induced sustained remission in 20 of 24 (83.3%) patients with complicated SDNS [17]. Kamei et al. and Fujinaga et al. also reported that a single dose of rituximab treatment was effective for children with complicated FRNS/SDNS [18, 19]. Ravani et al. reported a relatively large case series of patients who received one to five doses of rituximab as treatment for complicated SDNS (*n* = 46) [20]. In this series, 6-month probabilities of remission were 48% after the first infusion and 37% after subsequent infusions, and 1- and 2-year remission probabilities were 20 and 10%, respectively. The time to reconstitution of CD20 cells correlated with the duration of remission. Five patients required rituximab infusion in the intensive care unit for initial bronchospasm, which improved after slowing the infusion rate. Two patients had neutropenia associated with transient viral infection [20]. Webb et al. carried out a retrospective cohort study on 102 children who were treated with cyclophosphamide and/or rituximab [21]. These authors found that rituximab was associated with a longer remission time and fewer side effects than cyclophosphamide.

Members of the International Pediatric Nephrology Association were asked to retrospectively complete a questionnaire describing the use of rituximab in their respective center [22]. In this survey, 28 patients with complicated FRNS/SDNS were treated with one to four doses of rituximab, of whom 23 (82%) had a good initial response.

To summarize, the results of all these case reports, case series and survey studies are promising.

## RCTs of rituximab for complicated FRNS/SDNS

### An open-label RCT (non-inferiority test)

Ravani et al. conducted the first RCT of rituximab for children with steroid- and calcineurin-dependent nephrotic syndrome (i.e., complicated SDNS). This trial was an open-label RCT that examined the short-term effects of rituximab in children with complicated SDNS [23]. This trial was a non-inferiority test in which the primary efficacy measure was the percentage change in daily proteinuria at 3 months in children receiving rituximab (one dose) versus those on standard care. In this trial, 54 children were randomized (*n* = 27 in each group). The results showed that proteinuria at 3 months after treatment was 70% lower in the rituximab arm [95% confidence interval (CI) 35–86%] than in the standard therapy arm. The relapse rates were 18.5% (intervention) and 48.1% (standard arm) (*p* = 0.029), and the probabilities of being drug-free at 3 months were 62.9 and 3.7% (*p* < 0.001), respectively. Additionally, at 6 and 12 months of follow-up, 50 and 25% of children who were assigned to the rituximab arm were still in remission without prednisone or calcineurin inhibitor therapy, respectively. This study shows that rituximab and low doses of prednisone and calcineurin inhibitors are as efficacious as standard therapy in terms of maintaining short-term remission in children with idiopathic nephrotic syndrome who are dependent on both drugs and allow their temporary withdrawal.

### A multicenter, double-blind, randomized placebo-controlled trial

From 2008 to 2011, the Research Group of Childhood-onset Refractory Nephrotic Syndrome (RCRNS) in Japan conducted a multicenter, double-blind, randomized, placebo-controlled trial (RCRNS01; Clinical Trials Registry ID: UMIN000001405) [11] which evaluated the efficacy and safety of rituximab in childhood-onset complicated FRNS or SDNS. RCRNS01 was an investigator-initiated clinical trial whose aim was to gain official approval from the Japanese government for the use of rituximab for patients with childhood-onset complicated FRNS/SDNS. Because the use of rituximab for the treatment of nephrotic syndrome had not been approved in any country at that time, the RCRNS adopted a gold-standard, double-blind, placebo-controlled trial design, with as much consideration for the placebo group as possible. In this trial, treatment failure was defined, and if patients had failure of treatment, the allocation code was immediately disclosed. If patients were allocated to the placebo group, they were able to choose to begin the optimal treatment as determined by investigators (e.g. administration of new immunosuppressive drugs) and to continue this study or to enter a separately conducted rituximab pharmacokinetic study.

Patients who had a relapse of nephrotic syndrome were treated with protocol-defined prednisolone therapy and underwent screening examinations. Investigators and patients were blinded to peripheral B-cell counts, which were centrally monitored. Once patient eligibility, including steroid sensitivity, was verified, patients were randomly assigned (1:1) to one of two treatment groups. The rituximab group received 375 mg/m^2^ body surface area of intravenous rituximab (maximum 500 mg) once weekly for 4 weeks. The placebo group received placebo at the same frequency. After remission was achieved, prednisolone and immunosuppressive drugs were gradually tapered, and patients were followed up for 1 year.

The primary endpoint was the relapse-free period, which was defined as the time of randomization to the time of the first relapse after the start of the study treatment. Adverse events, including infection, were also evaluated.

**Fig. 1:**
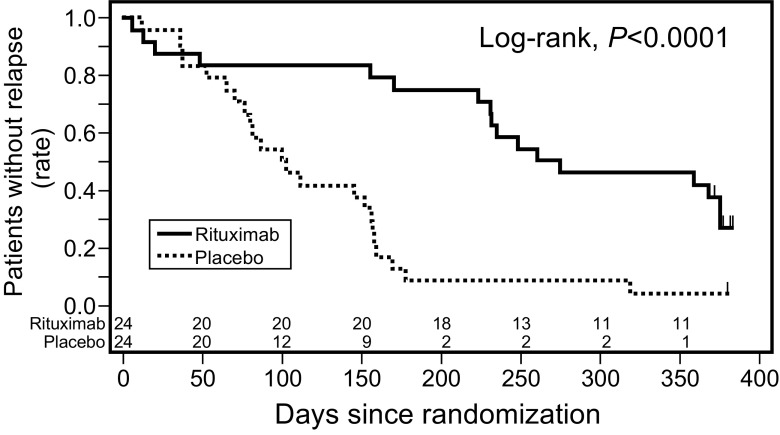
Relapse-free survival probability in the trial conducted by the Research Group of Childhood-onset Refractory Nephrotic Syndrome (RCRNS)

A total of 63 patients were screened, and 52 were randomized, with 27 in the rituximab group and 25 in the placebo group. Twenty-four patients in each group (total: 48) received the intervention and were included in the intention-to-treat analysis. The 50% relapse-free period was significantly longer in the rituximab group than in the placebo group [267 vs. 101 days; hazard ratio (HR) 0.267, 95% CI 0.135–0.528; *p* < 0.0001) (Fig. 1). The relapse rate was significantly lower in the rituximab group than in the placebo group [1.542 (29/18.81) vs. 4.171 (46/11.03) person-years; HR 0.370, 95% CI 0.231–0.591; *p* < 0.0001). The daily steroid dose after randomization was significantly lower in the rituximab group than in the placebo group (9.12 ± 5.88 vs. 20.85 ± 9.28 mg/m^2^/day; *p* < 0.0001). In this trial, no deaths were reported and the majority of adverse events were mild. The rate of serious adverse events, including gastroenteritis, cellulitis, neutropenia, gum infection, respiratory disturbance and hypoproteinemia [42% (10/24) vs. 25% (6/24); Fisher’s exact test, *p* = 0.3587], and infusion reaction [79% (19/24) vs. 54% (13/24); Fisher’s exact test, *p* = 0.1246] were similar in the two groups. No patients in either group experienced Grade 3 or 4 infusion reactions. These findings indicate that rituximab is a safe and effective therapy, at least for 1 year, for childhood-onset, complicated FRNS or SDNS.

### Approval of rituximab use and its effect on management of patients in Japan

Based on the results of the RCRNS trial, rituximab was approved by the Ministry of Health, Labor and Welfare of Japan for treating patients with complicated FRNS or SDNS on August 29, 2014. As such, from this date rituximab has been covered by the health insurance system in Japan for the treatment of these conditions. Just after this approval, an “all-case” surveillance study to confirm the safety and efficacy of rituximab administered to patients complicated by FRNS/SDNS in clinical practice was initiated in Japan. The observational period of the survey is 2 years. By April 15, 2016, 945 patients, including 425 children younger than 15 years of age, were enrolled into the survey study [24]. Re-evaluation results of the safety and efficacy of rituximab from a large number of patients will be obtained in the near future.

## RCTs of rituximab for uncomplicated SDNS

A long-term follow-up study showed that drug-free remission after rituximab treatment tended to last longer in children who were treated with steroids and calcineurin inhibitors, who were initially dependent on steroids alone and who had a shorter disease duration [20]. Therefore, Ravani et al. conducted a multicenter, open-label RCT in children with early-stage uncomplicated SDNS [25]. These children had normal levels of proteinuria, and their state of complete remission was dependent on treatment with high-dose steroids alone for 6–12 months. The aim of this trial was to determine whether rituximab is non-inferior to steroids in terms of maintaining complete remission.

In this study, participants were randomly assigned to continue prednisone alone for 1 month (control group) or to have a single intravenous infusion of rituximab added to the therapy (375 mg/m^2^; intervention group) [25]. Steroids were tapered to determine whether a single pulse of rituximab allows complete steroid withdrawal and maintains steroid-free remission. The primary outcome was the percentage change in daily proteinuria at 3 months of follow-up. In this trial, 30 children were randomized (1:1) and included in the analysis on an intention-to-treat basis. The results showed that proteinuria at 3 months of follow-up was 42% lower in the intervention group (prednisone + rituximab) than in the control group (only prednisone) and that rituximab was non-inferior to prednisone as treatment. Additionally, the 1-year relapse-free survival rate in the intervention group was significantly higher than that in the control group (66 vs. 0%; *p* < 0.01). However, the 2-year relapse-free survival rate in the intervention group was not satisfactory (34%) [25].

During rituximab infusion, all of the participants developed a mild infusion reaction (nausea and/or skin rash). This reaction was successfully treated in all cases by slowing the infusion rate and increasing the dose of chlorfenamine [25]. One participant had fever, skin rash and acute hip joint arthritis 32 days after rituximab treatment. However, these symptoms rapidly resolved by nonsteroidal anti-inflammatory medications.

The results of this study show that rituximab is non-inferior to steroids for treating the uncomplicated form of SDNS. They also suggest the possibility of rituximab as the first-line treatment for SDNS.

## Adverse events of rituximab in SSNS in children

Rituximab is associated with several serious adverse events, including fatal hepatitis induced by rituximab reactivation of hepatitis B virus [26] and progressive multifocal leukoencephalopathy [27]. Rituximab is also associated with serious adverse events in children with complicated FRNS/SDNS, including pulmonary fibrosis [28], fulminant myocarditis [29], pneumocystis pneumonia [16], immune-mediated ulcerative colitis [30] and agranulocytosis [31]. After rituximab treatment, hypogammaglobulinemia persists in most patients with decreased immunoglobulin G (IgG) levels before treatment [32]. Recently, two patients developed hypersensitivity reactions, including anti-rituximab antibodies, during a second course of rituximab infusion [33]. In one study, the 3-year mortality rate following initiation of anti-CD20 therapy in patients with various autoimmune diseases was reported to be 3%, with most deaths due to infection [34]. Additionally, impaired T-cell immunosurveillance due to B-cell depletion by rituximab may cause secondary malignancy, such as melanoma [35, 36] or other skin tumors [37]. Rituximab-included chemotherapy for lymphoma may be a risk factor for secondary solid tumors [38]. Although a recent meta-analysis showed that the use of rituximab does not increase secondary malignancy [39], patients who receive rituximab must be followed up for a long period.

## Future perspectives

Almost all patients whose steroid and other immunosuppressive therapies are withdrawn after rituximab treatment have relapses after recovery of peripheral B-cell counts. Therefore, further modification of rituximab treatment, including repeated courses of rituximab and adjunct immunosuppressive therapies, may be necessary for maintaining long-term remission. Sellier-Leclerc et al. reported a case series which suggested that repeated rituximab infusions after B-cell recovery were effective for maintaining long-term remission in patients with complicated FRNS/SDNS [40]. Kimata et al. reported a case series which showed that rituximab administration for four times at 3-month intervals induced long-term remission without serious adverse events in children with complicated SDNS [41]. However, the effect of persistent B-cell depletion on the developing immune system in children is unknown. Additionally, poor efficacy of vaccination under persistent B-cell depletion is a serious problem in children.

A case series by Ito et al. suggested that maintenance therapy with MMF after rituximab administration was effective for maintaining long-term remission in children with complicated FRNS/SDNS [42]. These findings led to a multicenter, double-blind, randomized, placebo-controlled trial (JSKDC07; Clinical Trials Registry ID: UMIN000014347) being conducted by the Japanese Study Group of Kidney Disease in Children (JSKDC). The aim of this trial was to assess the efficacy and safety of MMF after rituximab treatment in children with complicated FRNS/SDNS.

The efficacy, safety and cost-effectiveness of various rituximab dosing regimens should be compared to determine an appropriate rituximab treatment regimen for complicated FRNS/SDNS in children. In one retrospective cohort study, the time to first relapse was significantly shorter in patients who received one to two doses of initial rituximab infusion compared with those who received three to four doses [43]. However, the proportion of patients with long-term remission was not related to the number of initial rituximab applications [43]. Large-scale multicenter cohort studies or multicenter RCTs to compare treatment outcomes after different dosing regimens are required to clarify the optimal dosage of rituximab to use.

Rituximab can be used as a first-line drug to treat cases of uncomplicated SDNS. Indeed, rituximab has been used as a first-line treatment for uncomplicated SDNS in many centers in European countries [44], but further studies are required to clarify its efficacy and safety for such cases. A relatively large-scale, open-label, parallel-arm, controlled trial comparing the efficacy and safety of rituximab with that of tacrolimus in children with uncomplicated SDNS is currently being conducted in India (Clinical Trials Registry ID: NCT02438982). The results of this study will deserve attention.

## Conclusions

Rituximab is a promising treatment for complicated FRNS/SDNS and uncomplicated forms of SDNS. However, further studies are required to establish optimal treatment protocols for each condition. Additionally, long-term prognosis, including the risk of developing malignancy, should be clarified.
